# Attentional modulation of interhemispheric (a)symmetry in children with developmental language disorder

**DOI:** 10.1038/s41598-022-22820-x

**Published:** 2022-10-25

**Authors:** Doris Hernández, Salme Kärkkäinen, Terhi Tulonen, Päivi Helenius, Riitta Salmelin, Tiina Parviainen

**Affiliations:** 1 Center for Interdisciplinary Brain Research, Department of Psychology, University of Jyväskylä, Kärki, Mattilanniemi 6, P.O. Box 35, 40014 Jyväskylä, Finland; 2 Department of Mathematics and Statistics, University of Jyväskylä, P.O. Box 35, 40014 Jyväskylä, Finland; 3 Division of Child Neurology, Helsinki University Hospital, HUS, P.O. Box 100, 00029 Helsinki, Finland; 4 Department of Neuroscience and Biomedical Engineering, Aalto University, P.O. Box 12200, 00076 Espoo, Finland; 5 Aalto NeuroImaging, Aalto University, P.O. Box 15100, 00076 Espoo, Finland

**Keywords:** Neuroscience, Psychology

## Abstract

The nature of auditory processing problems in children with developmental language disorder (DLD) is still poorly understood. Much research has been devoted to determining the extent to which DLD is associated with general auditory versus language-specific dysfunction. However, less emphasis has been given to the role of different task conditions in these dysfunctions. We explored whether children with DLD demonstrate atypical interhemispheric asymmetry during the auditory processing of speech and non-speech sounds and whether this interhemispheric balance is modulated by attention. Magnetoencephalography was used to record auditory evoked fields in 18 children (9 to 10 years old), 9 with DLD and 9 with language typical development, during active or passive listening to speech and non-speech sounds. A linear mixed model analysis revealed a bilateral effect of attention in both groups. Participants with DLD demonstrated atypical interhemispheric asymmetry, specifically in the later (185–600 ms) time window but only during the passive listening condition. During the active task, the DLD group did not differ from the typically developed children in terms of hemispheric balance of activation. Our results support the idea of an altered interhemispheric balance in passive auditory response properties in DLD. We further suggest that an active task condition, or top–down attention, can help to regain leftward lateralization, particularly in a later stage of activation. Our study highlights the highly dynamic and interhemispheric nature of auditory processing, which may contribute to the variability in reports of auditory language processing deficits in DLD.

## Introduction

Developmental language disorder (DLD), also known as specific language impairment (SLI), has been linked to the abnormal processing of auditory information^1,2^. The main deficit in children with DLD has been suggested to be related to the processing of speech sounds^3–6^, with influences at different levels such as vowels^5^, syllables^3,4,6^, words^7,8^, and longer narratives^9,10^. However, others claim that impairment arises at a more basic level of auditory processing, such as the processing of non-speech sounds^11^ or all kinds of sounds, including speech and non-speech, which would expand the deficit to a more generalized auditory processing impairment^2^. Given the dynamically varying and task-specific nature of auditory language processing, it is likely that multiple factors, which may vary across experiments, contribute to the specific expression of the deficit at the neural level. In the current study, instead of approaching the deficit in DLD based on the types of sounds, we take a more general approach, focusing on the role of attention (namely, active versus passive task conditions) to the auditory processing of different (speech, non-speech) sound types.

In the study of auditory and language perception in DLD, little consideration has been devoted to the so-called central functions that may modulate language perception and higher cognitive processes during language acquisition and that can have a major impact on the appearance of the functional deficit. Indeed, some evidence suggests that attention and its selectivity may be involved in the perceptual problems observed in DLD^5,9,10,12^. With the use of event-related potentials (ERPs), children with DLD have shown a different pattern of activation during attention toward speech sounds compared with typically developing children. When they were asked to attend to tones embedded in speech sounds, both groups of children showed an equivalent level of involuntary attention to the speech sounds in the active task condition. However, in the passive task, children with DLD allocated delayed and less emphasized neural resources to processing speech sounds than did typically developing children^5^. Using behavioral tasks, Niemi et al.^9^ showed difficulties in modifying the ear advantage through focused attention to the left ear in children with DLD. Even though attention has shown a modulatory influence on auditory processing in children with DLD, its contribution is still not clear, as some studies have also found no effects of attention in children with DLD^13,14^.

The earlier results also reveal another important source of discrepancy in previous studies of DLD, namely the question about the hemispheric lateralization of the deficit. The literature has shown diverse patterns of impaired hemispheric involvement in auditory processing in children with DLD. The deficit is suggested to be left dominant^8,9,11,15–17^, right dominant^18,19^, or bilateral^20^. Thus, it is unclear to what extent the problems in DLD should be approached as hemisphere-specific or interhemispheric interaction problems. Based on the complexity of language processing, a correct balance between the brain hemispheres, more than the specific involvement of each of them, is likely to be required^21^, and there may also be developmental differences in lateralization in typical development^22,23^. Structurally, atypical interhemispheric asymmetry has been observed in children with DLD. Using structural MRI, Plante et al.^24^ showed larger right than left perisylvian areas in children with DLD than in control children.

Approaching the main deficit of DLD as interhemispheric asymmetry rather than a specific hemispheric problem could help to better understand the behavioral manifestations of the disorder. Indeed, by considering both brain hemispheres and their interaction, some discrepancies in the literature regarding hemispheric deficits in DLD could be explained [see, e.g.,^8,9,11,15–20^].

Due to the interhemispheric connections and functional lateralization of auditory perception, the question of interhemispheric asymmetry and its attentional modulation is closely intertwined with the nature of auditory processing deficit. In general, leftward lateralization is typically reported for processing speech sounds^25,26^, but as stated above, the picture is less clear with respect to the lateralization of the deficit in DLD, both for speech and for nonspeech sounds. Importantly, regarding the nature of the auditory processing deficit, it is not clear what the role of attention (i.e., active versus passive listening conditions) is in abnormal lateralization or interhemispheric balance in DLD. To clarify this in the present study, we tested the effect of attentional modulation on the auditory processing of speech and non-speech sounds in the two hemispheres of children with DLD.

With time-sensitive imaging techniques, such as electroencephalography (EEG) and magnetoencephalography (MEG), it is possible to track the sequence of neural processing from early sensory cortices to later perceptual activation and beyond. MEG also allows us to reliably examine the interhemispheric balance in activation. Some studies have reported abnormal auditory activation in DLD during the first stages of auditory processing, occurring within 100 ms post-stimulation^2,4,5,10,18^, generally identified as reflecting essentially sensory processing of information. In this time window, the deficits described are related to delayed^18^ and weaker^2,4,5,10^ auditory perceptual processing in children with DLD. Other studies allocate the deficit to a later time window, 200–600 ms after stimulus presentation^6,8,11,17,27^, comprising pre-lexical, phonological, and lexical-semantic processes. The deficits identified in this later time window were related to the short-term maintenance of linguistic activation that underlies spoken word recognition^6,8^, frequency discrimination^11,27^, or the passive processing of tones^17^. The later time window is of particular interest for typical and atypical development because it shows a response type to purely passive stimulation, which is unique to the child’s brain^22,28–30^. It is noteworthy that most of the aforementioned studies were performed with EEG, where the fairly low spatial resolution does not allow us to reliably approach the hemispheric difference in activation^2,5,6,10,11,27^, or with MRI/fMRI, with limited temporal resolution for following the different stages of processing^15,20,24^.

Here, we used MEG to clarify interhemispheric asymmetry during attended and non-attended auditory processing in DLD across time. The neural activation derived from the MEG recordings in the left and right hemispheres of children with DLD and typically developing children was compared during the active and passive listening of speech and non-speech sounds. Specifically, we tested the hypothesis that (1) children with DLD show abnormal interhemispheric asymmetry during the auditory processing of speech and non-speech sounds. Furthermore, based on earlier findings^5,9,10,12^, we hypothesized that (2) attention modulates interhemispheric asymmetry. We anticipated that the difference in the (interhemispheric) pattern of activation between children with DLD and children with typical language development would be smaller during active listening than during passive listening [cf.^5^]. To provide a novel understanding of the neural basis of auditory processing deficits in DLD, we further tested 3) whether the presumed abnormal interhemispheric asymmetry and the attentional modulatory effect occurred during early sensory processing or later stages of auditory processing. Particularly interesting in this respect is the prolonged activation evidenced predominantly in children^22^.

## Results

First time window (90–180 ms).

### Modeling

The model was built to test our research questions concerning (1) the abnormal interhemispheric (a)symmetry in DLD during the auditory processing of speech and nonspeech sounds and (2) the attentional modulation of this interhemispheric (a)symmetry. Using the backward method, all pairwise interactions were tested with a likelihood ratio test after testing that the three-wise interaction was not needed (chi^2^ = 0.006, *p* = 0.936, *df* = 1). In the reference model, we had a random intercept and a random slope for the hemisphere. The pairwise interaction group * attention was first removed (chi^2^ = 0.097, *p* = 0.755, *df* = 1). The interactions between hemisphere * attention (chi^2^ = 4.319, *p* = 0.038, *df* = 1) and hemisphere * group (chi^2^ = 1.474, *p* = 0.225, *df* = 1) were then tested separately and included in the model to directly test our research questions. Besides a random intercept, a random slope for the hemisphere (chi^2^ = 38.154, *p* < 0.001, *df* = 2) and their covariance (chi^2^ = 4.360, *p* = 0.037, *df* = 1) were required.

We also tested whether a random slope for attention with covariances was needed in the random part (besides the hemisphere) (chi^2^ = 3.240, *p* = 0.357, *df* = 3), and we did not include it in the final model. As we had a random slope for the hemisphere, no different variances for groups (chi^2^ = 0.447, *p* = 0.504, *df* = 1) were needed. We additionally pre-studied the models, including sound and a random intercept, and found that the pairwise interactions could be removed from the model in the following order: sound type * group (chi^2^ = 0.078, *p* = 0.962, *df* = 2), group * attention (chi^2^ = 0.077, *p* = 0.782, *df* = 1), sound type * attention (chi^2^ = 0.944, *p* = 0.624, *df* = 2), and hemisphere * sound type (chi^2^ = 1.295, *p* = 0.523, *df* = 2), leaving only sound in the model (chi^2^ = 22.127, *p* < 0.001, *df* = 2). To have more power to study a random part and our pre-specified research questions, we continued modeling without sound and resulted with a model with the main effects group, attention, and hemisphere, two interactions (hemisphere * attention and hemisphere * group), and a random part with the intercept, the slope for the hemisphere, and their covariance.

The final model obtained from the mixed-model approach for the first time window (90–180 ms) is presented in Table 1, showing the estimates, standard errors, their ratios (t-values), and their *p*-values. The estimates and confidence intervals for the standard deviations of the random intercept, the random slope along with their correlation, and the residual are shown in Table 2. All the assumptions (linearity, variance structure, and normality) for the model were sufficiently fulfilled.

**Table 1:** Fixed effects of the model for the first time window: estimated difference (standard error [SE]), degrees of freedom, t-value, and *p*-value.

	Est. value (SE)	*df*	t-value	*p*-value
Intercept	35.83 (7.60)	188	4.713	0.000
Hemisphere^a^	− 6.92 (6.21)	188	− 1.115	0.266
Group^b^	2.12 (10.62)	16	0.199	0.845
Attention^c^	9.58 (2.45)	188	3.917	0.000
Hemisphere × group	9.89 (8.47)	188	1.168	0.244
Hemisphere × attention	7.18 (3.45)	188	2.080	0.039

^a^Baseline for hemisphere: right, ^b^baseline for group: DLD, and ^c^ baseline for attention: passive.

**Table 2:** Approximate 95% confidence intervals for the standard deviations of a random intercept, a random slope for hemisphere, and the correlation between the random intercept and the random slope, and for the standard deviation of a residual.

	Lower	Est	Upper
Intercept	15.22	21.93	31.60
Hemisphere	10.85	16.40	24.79
Cor (int,hemi)	− 0.81	− 0.53	− 0.04
Residual	11.135	12.37	13.75

### Interhemispheric asymmetry and effects of attention

**Figure 1:**
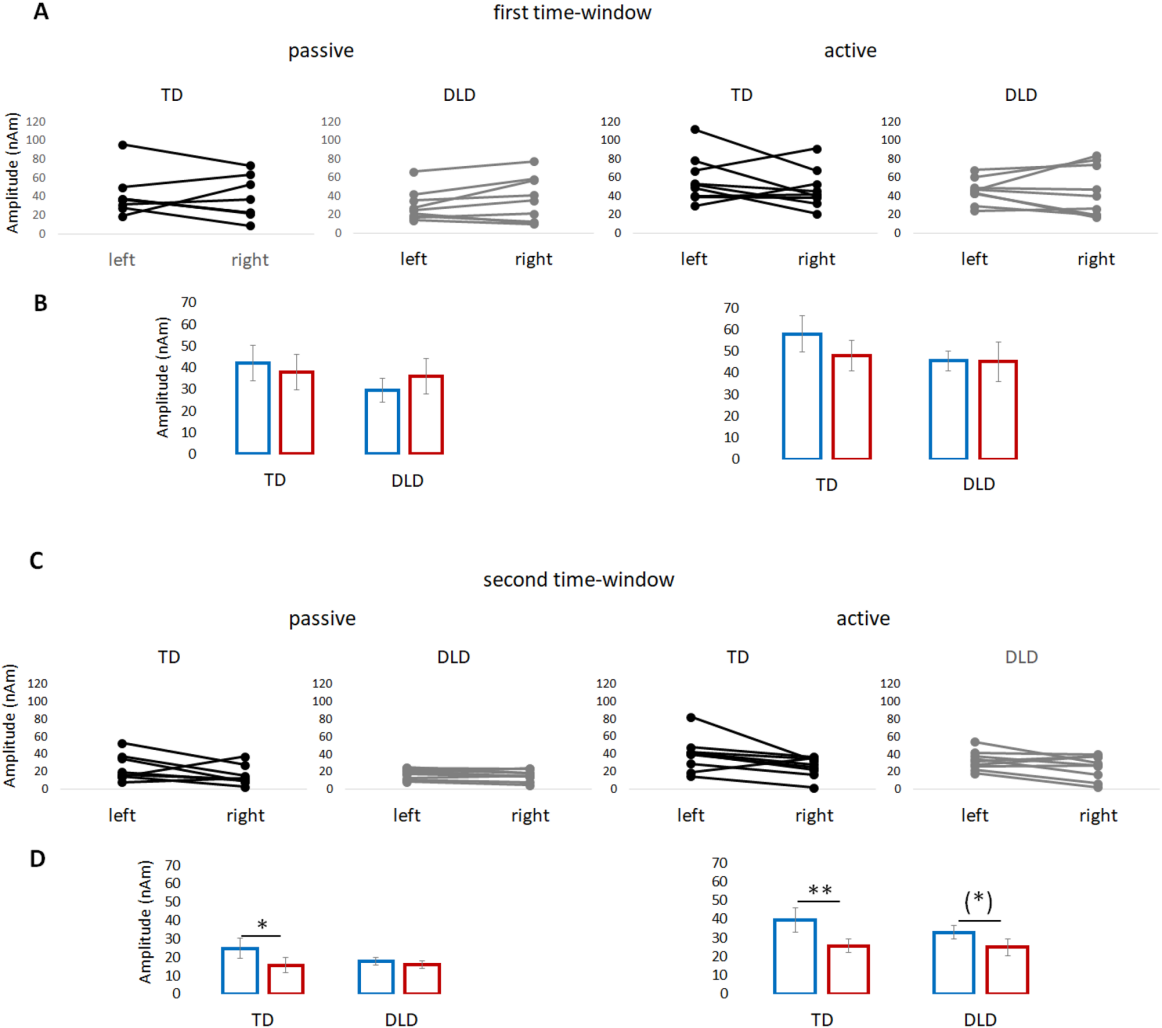
Main results in both time windows. (**A**) Comparison of the individual strength of activation between the left and right hemispheres in response to passive and active conditions in the first time window for children with DLD (gray) and children with TD (black). (**B**) Comparison of the averaged activation between the left (blue) and right (red) hemispheres in response to active and passive conditions in the first time window for children with DLD and children with TD. (**C**) Comparison of the individual strengths of activation between the left and right hemispheres in response to passive and active conditions in the second time window for children with DLD (gray) and children with TD (black). (**D**) Comparison of the averaged activation between the left (blue) and right (red) hemispheres in response to active and passive conditions in the second time window for children with DLD and children with TD. Whiskers represent the standard error of the mean (SEM).

Figure 1A shows the individual amplitude values for the first time window separately for the TD group and the DLD group. The average amplitude values of responses from the data per group and condition for the first time window are depicted in Fig. 1B. The results outlined above are based on the fitted random intercept model, and they describe the differences in the raw average values well (Fig. 1B).

Attention showed a clear main effect, with generally higher amplitudes in the active condition than in the passive condition (ED = 9.58, SE = 2.45, t(188) = 3.917, *p* = 0.001). There was also a significant interaction between attention and hemisphere (ED = 7.18, SE = 3.45, t(188) = 2.080, *p* = 0.039).

Consequently, we further tested hemispheric balance under different attentional conditions, separately in the DLD and TD groups, to directly evaluate our hypothesis on the divergent hemispheric asymmetry of activation in DLD versus TD. In the TD group, the amplitude in the left versus right hemispheres did not differ in the passive (ED = 2.97, SE = 6.30; t(188) = 0.471, *p* = 0.638) or active conditions (ED = 10.15, SE = 6.21; t(188) = 1.635, *p* = 0.104). In the DLD group, there was also no difference between the left and right hemispheres, whether in the passive (ED =  − 6.92, SE = 6.21; t(188) =  − 1.115, *p* = 0.266) or active conditions (ED = 0.25, SE = 6.21; t(188) = 0.041 *p* = 0.967). Figure 3 shows the grand average waveforms for both groups (children with DLD and TD) for the left and right hemispheres during the active and passive conditions.

Second time window (185–600 ms).

### Modeling

The model was built to test the possible group differences in hemispheric (a)symmetry during auditory processing of speech and non-speech sounds, as well as the attentional modulation of this interhemispheric asymmetry. As in the first time window, all the pairwise interactions were tested with a likelihood ratio test after testing and removing the three-wise interactions (chi^2^ = 0.103, *p* = 0.748, *df* = 1). In the reference model, we had a random intercept and random slopes for the hemisphere and attention and their covariances. The covariates and their interactions to be tested were group, hemisphere, and attention. The pairwise interaction group * attention was removed (chi^2^ = 0.002, *p* = 0.968, *df* = 1). At the next level, the pairwise interaction group * hemisphere was kept in the model (chi^2^ = 1.510, *p* = 0.219, *df* = 1) to test our specific research questions. Moreover, we checked the random part of the previous model such that different variances for the groups were added. Besides attention, the hemisphere was required (chi^2^ = 25.274, *p* < 0.001, *df* = 3), and besides the hemisphere, attention was needed (chi^2^ = 17.614, *p* < 0.001, *df* = 3). The interaction between hemisphere and attention was not required (chi^2^ = 1.732, *p* = 0.279, *df* = 1). Moreover, the off-diagonal terms in the covariance matrix for a random part were tested and not needed (chi^2^ = 2.392, *p* = 0.495, *df* = 3). The difference in the variances in groups was not significant for the mixed model with two interaction terms at the population level and the random intercept and slopes for hemisphere and attention at the individual level (chi^2^ = 0.866, *p* = 0.352, *df* = 1).

We also tested the pairwise interactions for sound and attention, hemisphere, and group with a random intercept: attention * group (chi^2^ = 0.003, *p* = 0.955, *df* = 1), attention * sound type (chi^2^ = 1.071, *p* = 0.585, *df* = 2), hemisphere * sound type (chi^2^ = 1.976, *p* = 0.374, *df* = 2), and group * sound type (chi^2^ = 3.110, *p* = 0.211, *df* = 2). After testing the pairwise interactions, we also tested the significance of the sound type (chi^2^ = 28.685, *p* < 0.001, *df* = 2). Due to statistical power, the sound type was not included in the more precise model selection because it did not show any interactions.

The final model for the second time window (185–600 ms) is presented in Table 3, which shows the estimates, standard errors, their ratios (t-values), and their *p*-values. The estimates and confidence intervals for the standard deviations of the random intercept and random slopes, along with the residual, are shown in Table 4. All the assumptions for the model were fulfilled sufficiently well.

**Table 3:** Fixed effects of the model for the second time window: estimate (SE), degrees of freedom, t-value, and *p*-value.

	Est. value (SE)	*df*	t-value	*p*-value
Intercept	15.23 (3.09)	188	4.928	0.000
Hemisphere^a^	2.17 (4.01)	188	0.541	0.589
Group^b^	0.54 (4.32)	16	0.124	0.903
Attention^c^	9.87 (2.70)	188	3.662	0.000
Hemisphere × group	6.64 (5.42)	188	1.226	0.222
Hemisphere × attention	5.37 (2.48)	188	2.167	0.032

^a^Baseline for hemisphere: right, ^b^baseline for group: DLD, and ^c^baseline for attention: passive.

**Table 4:** Approximate 95% confidence intervals for the standard deviation of a random intercept, random slopes for attention and hemisphere, and a residual.

	Lower	Est	Upper
Intercept	5.11	7.97	12.43
Attention	5.49	8.61	13.50
Hemisphere	6.64	10.21	15.70
Residual	7.96	8.90	9.95

### Interhemispheric asymmetry and effects of attention

Attention showed a clear effect with higher amplitudes in the active than passive listening condition (ED = 9.87, SE = 2.70, t(188) = 3.662, *p* < 0.001). There was also a significant interaction between attention and hemisphere (ED = 5.37, SE = 2.48, t(188) = 2.167, *p* = 0.032). We tested hemispheric balance in the two attentional conditions, keeping the groups separate to directly test our research questions. The results revealed different patterns of interhemispheric asymmetry in the two groups (see Fig. 1). For the TD group, both attentional conditions indicated a higher mean amplitude in the left hemisphere than in the right hemisphere (passive condition: ED = 8.81, SE = 4.08; t(188) = 2.160, *p* = 0.032; active condition: ED = 14.18, SE = 4.01; t(188) = 3.539, *p* < 0.001). For the DLD group, there was no difference in amplitude between hemispheres in the passive condition (ED = 2.17, SE = 4.01; t(188) = 0.541, *p* = 0.589). However, in the active condition, the left hemisphere showed a higher mean amplitude than the right hemisphere, with a *p*-value close to significance (ED = 7.54, SE = 4.01; t(188) = 1.882, *p* = 0.061).

Figure 1C shows the individual amplitude values for the second time window. The average amplitude values of the responses from the data per group and condition for the second time window are depicted in Fig. 1D. The results outlined above are based on the fitted random intercept model, and they describe the differences in the raw average values well (Fig. 1D).

## Discussion

In this study, the hemispheric balance of auditory activation during active and passive listening was compared between groups of children with DLD and children with typical language development. Speech and non-speech sounds were used as stimuli, but sound type did not show any significant interaction with other factors, so it was not included in the final model. We followed the activation elicited by sounds in two separate time windows: early activation before 200 ms and long-lasting activation after 200 ms. Attention showed a clear effect on auditory activation over the entire time window and all sound types alike for both groups. The results revealed an atypical interhemispheric asymmetry of activation for the participants with DLD during passive listening, confirming our first hypothesis. More specifically, in the later prolonged activation, the DLD group demonstrated a relative decrease in the left compared to the right supratemporal area, independent of sound type. Interestingly, in the active condition, the interhemispheric balance shifted toward the direction observed in the TD group. This finding supports our second hypothesis that attention modulates interhemispheric asymmetry in DLD. An active mode of perception thus seems to play a significant role in the neural-level dysfunctions observed in DLD.

The atypical hemispheric balance observed in the DLD group during passive listening was significant in the later sustained activation. The relative involvement of the left hemisphere was smaller in the DLD group, with symmetrical activation of the left and right hemispheres, than in the TD group, with stronger responses in the left than in the right hemisphere. The atypical pattern of interhemispheric asymmetry during auditory processing could be interpreted as reflecting a hard-coded dysfunction in the neural basis of auditory perceptual processing in DLD, as was evident in the passive condition. This result is in line with previous studies showing differences between children with DLD and typically developed children in passive listening tasks^2,5,17^.

The hemispheric balance in the first time window did not indicate clear group differences. Shafer et al. (2007)^5^ reported that children with DLD showed a leftward asymmetry of the scalp distribution of the maximum of the peak studied (negative difference, Nd, in the latency range of N1) during passive listening to speech sounds, while typically developed children showed a rightward asymmetry. The inconsistency between their study and our results could, in principle, arise from differences in methods. Shafer et al.^5^ used EEG and topographic information of a fronto-central positivity and inferior-posterior negativity, generally considered to be consistent with sources in the left and right auditory cortices. We analyzed the MEG-derived waveforms of sources determined to be located directly in the auditory cortices.

However, it is also likely that the specific developmental stage has a major impact on activation at around 100 ms in children, and the age range of Shafer’s sample is less restricted (8 to 10 years old) than the children included in our study (9 to 10 years old). Two overlapping activation types are reported in this time window, with strong interindividual variability in their appearance^22^. More specifically, an early peak denoted as P1/P1m shows clearly delayed latencies in children and appears at around 100 ms^31^, overlapping in latency with the emerging N1/N1m response especially in younger individuals. Even in adults, these two responses show divergent hemispheric distributions^26^. Thus, depending on whether they are analyzed separately or not in a study, the results may vary greatly, which complicates the interpretations of activation (and comparison across studies) within this time window (90–180 ms) in children. In this case, a restricted age range (like in the case of our study), is desirable given how rapidly auditory encoding neural activity changes throughout childhood.

In the later stage of activation, at around 250 ms, the children with DLD showed a divergent hemispheric balance of activation compared to the children with TD when the stimuli were passively presented. The DLD group demonstrated bilaterally symmetric activation, while the TD group showed stronger responses in the left hemisphere compared to the right hemisphere. Therefore, the obligatory response properties of the auditory cortices seem to deviate in language-impaired children. The passive response properties in this time window were also altered in our earlier study of DLD^17^, but the pattern was somewhat different. Although we used almost the same sample as van Bijnen et al. (2019)^17^, in contrast to our results, they reported an increase, rather than a decrease, in left-hemisphere activation in children with DLD. This suggests that not only the cortical auditory response properties but also the cortical appearance of language-related dysfunctions are highly sensitive to differences in stimulation conditions (monaurally presented simple tones in van Bijnen et al. (2019)^17^ versus binaurally presented mixture of speech, complex sounds, and tones in the current study).

**Figure 2:**
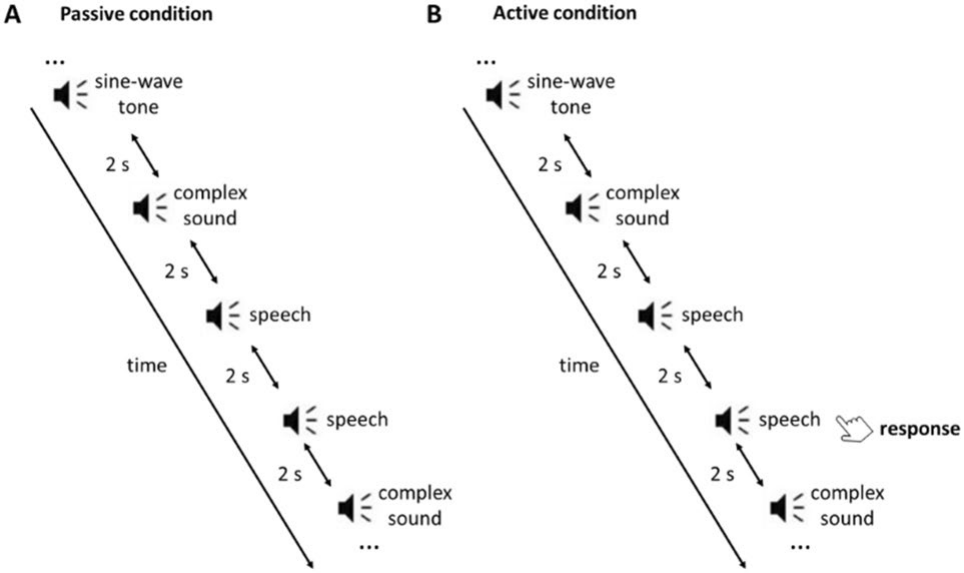
Schematic representation of passive and active conditions in the listening task for the passive (**A**) and active (**B**) conditions.

**Figure 3:**
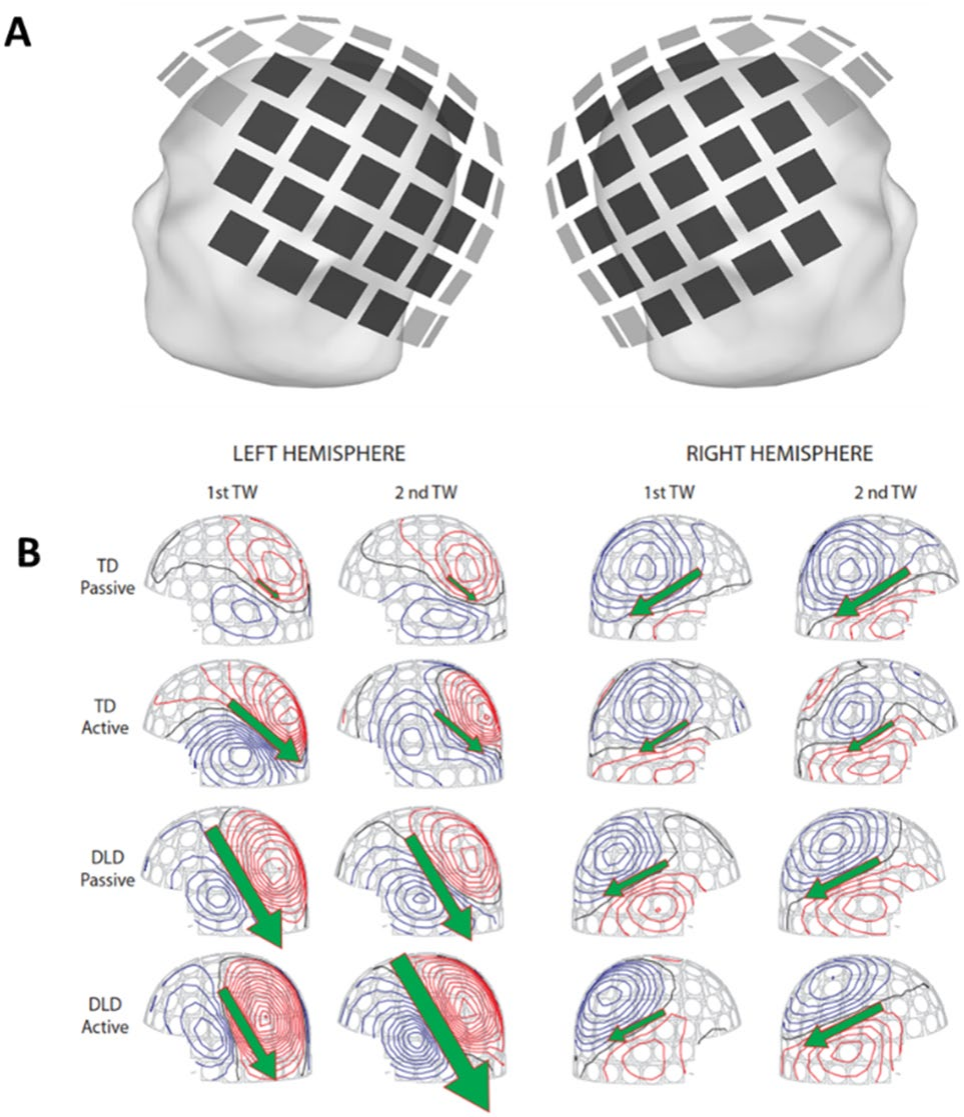
(**A**) Plot representing the 22 sensors included in the ECD model for each hemisphere. (**B**) Field distribution and ECD orientation (arrows) in the left and right hemispheres in both time windows of one subject in the TD group (above) and one subject in the DLD group (below) in active and passive conditions for speech sound.

It is also noteworthy that the activation evoked by all sound types in the present study (see Fig. 3) continues well after 600 ms, both for active and passive conditions, whereas the responses to the passive presentation of only simple sine-wave tones in van Bijnen et al. (2019; see Fig. 2 in that article) return to the baseline clearly earlier, by 500 ms at the latest. This salient difference in the persistence of activation in the perisylvian area, especially for the active condition, speaks to strongly contextual (and predictive) processing of sensory information.

Consequently, the expression of the deficit in DLD at the neural level may diverge across dissimilar experiments due to the dynamically varying and task-specific nature of auditory processing. This can be significantly pronounced when language processing is also involved. Indeed, the current results, together with van Bijnen et al. (2019)^17^, are compatible with the idea that the functional division of labor between the hemispheres in automatic responsivity to auditory (language) information may be compromised in DLD, with an atypical balance toward the left hemisphere in processing nonlinguistic (sinewave) stimuli and an atypical balance toward the right hemisphere in processing linguistic stimuli. It is important to note, however, that we did not see an interaction between sound type (speech versus nonspeech) and group in the present study; therefore, there is no support for language specificity of the auditory deficit in DLD. Nonetheless, given the above reasoning on the sensitivity of auditory processing to various factors, this may also reflect the strong influence of context on processing (presence of speech stimuli).

Previous investigations examining the potential brain structures implicated in DLD have also reported differential patterns of atypical brain organization involving both brain hemispheres^20,24,32,33^. Badcock et al. (2012)^20^ used MRI and fMRI to compare gray matter volumes and brain activation during the auditory processing of speech. The authors reported reduced gray matter volumes in the right caudate nucleus and increased gray matter volumes in the left inferior frontal cortex in children with DLD. Reduced gray matter volumes in children with DLD were also found bilaterally in the superior temporal cortex. Furthermore, during speech processing, they reported bilateral underactivation of the superior temporal cortex for children with DLD. Although the left and right hemispheres did not seem to differ in the latter area, the study demonstrated bilateral effects in children with DLD, in contrast to the one-hemisphere deficit often reported.

Similarly, Plante et al.^24^ showed atypical structural rightward perisylvian asymmetries (the right area was larger than expected, whereas the left area was of expected size) in children with DLD, with a link to their language performance. To date, several structural brain asymmetries have been reported; however, their associations with functional measures and behavioral manifestations of the disorder need to be better elucidated. More studies are needed to clarify the specific functional and behavioral consequences of a structural atypical hemispheric balance in DLD. Notably, however, these existing results align with our findings on the importance of focusing on interhemispheric balance rather than hemisphere-specific effects in understanding auditory deficits in DLD.

Besides reflecting abnormal (structural) wiring, the deviations in interhemispheric balance in DLD during passive listening could also indicate bias in automatic preference for speech versus other sound types. It has been reported that children with typical development pay attention to speech even when they are instructed to ignore it^5,34^. Thus, the pattern showing a leftward bias for all sounds in children with typical development^2^, also reported by the present study, could result from automatically emerging increased responsiveness of cortical processing toward speech sounds as a result of a developmental interplay between inherited and environmental factors. Consequently, the diminished responsiveness of the auditory cortex of children with DLD to speech could reflect atypical structural properties associated with the disorder, compromising the typical speech tuning of the cortex during development. Indeed, some studies suggest that developmental disorders of language, such as dyslexia, are associated with genetically regulated dysfunction at an early stage of development (migration)^35^, although this view has also been critically evaluated^36^.

The plausible diminished responsiveness of the auditory cortices in DLD might be suggesting that what is elicited by this type of task in children with DLD is a very delicate and small difference. This can be supported by the nature of the behavioral manifestations of DLD disorder. In the literature (and in our study) the auditory deficit in children with DLD are usually small ones. This is an important notion and might be suggesting paying attention to the small signs of developmental disorders to avoid leaving important signs unnoticed.

Interestingly, during active auditory processing, a deviant interhemispheric balance in children with DLD was not evident. Further, the DLD group did not differ from the TD group in the distribution of activation across the hemispheres and showed similar leftward lateralization. Thus, our results indicate that while passive response properties of the auditory cortices evidence an atypical left–right balance of activation, active engagement in the processing of the incoming auditory stream of sounds can modify the altered hemispheric balance.

In this study, we focused on the interhemispheric balance of brain activation during auditory processing, rather than on the specific involvement of each hemisphere in active and passive conditions. Such different approaches may explain the varying results regarding hemisphere-specific results in DLD. Our results are in line with the claims of Badcock et al.^20^ and Plante et al.^24^, who showed bilateral structural abnormalities in DLD. At the same time, our results do not disagree with studies showing either specifically left-hemisphere^8,9,11,15–17^ or right-hemisphere^18,19^ deficits in participants with DLD. Our present findings suggest that, for a better understanding of the deficit, it may be beneficial to focus on the relative involvement of both hemispheres. In general, when comparing conditions, relational measures across hemispheres may also be more reliable and informative due to the many (even structural) factors that may influence amplitude measures [cf.^37^].

The differential pattern of hemispheric balance across groups emerged at the later time window, which has earlier been indicated in the processing of phonetic and phonological information^38–40^, and thus may also relate here to a rather abstract level of analysis. However, processing any auditory information, including passively presented simple tones, seems to evoke activation in this later time window, specifically in children^17,41^. Thus, it seems likely that both the automatic responsivity of the auditory cortex (the so-called obligatory, exogenous elements) and active task-driven processes (endogenous elements) can underlie the deviant pattern of activity in DLD.

Most studies attributed the effect of attention in participants with DLD to an underlying deficit in attentional processes^5,9,10,12^. Our results suggest that active task conditions may mitigate the atypical (passive) response characteristics to sounds in DLD and do not imply a causal role for attentional deficit, at least when simple passive versus active task conditions are considered. Indeed, it is important to note that the active condition in the present study was merely required to process the identity of the incoming sound signal to identify immediate repetitions. This was done to avoid sound type-specific effects. Naturally, we cannot infer that our results are related to more complex attentional demands and their possible role in the behavioral-level outcomes of DLD.

As attention seems to modulate the involvement of the brain hemispheres during the auditory processing of sounds in children with DLD, this might open a new line of rehabilitation for DLD disorder. Strengthening general factors (like attention) that support the processing of sounds rather than the dysfunctional part (language processing), may prove valuable in dealing with children with DLD. Thus, using approaches that contribute to improving attentional capacity during developmental stages of life, could enhance the auditory processing, and furthermore, the behavioral manifestations of the disorder.

**Figure 4:**
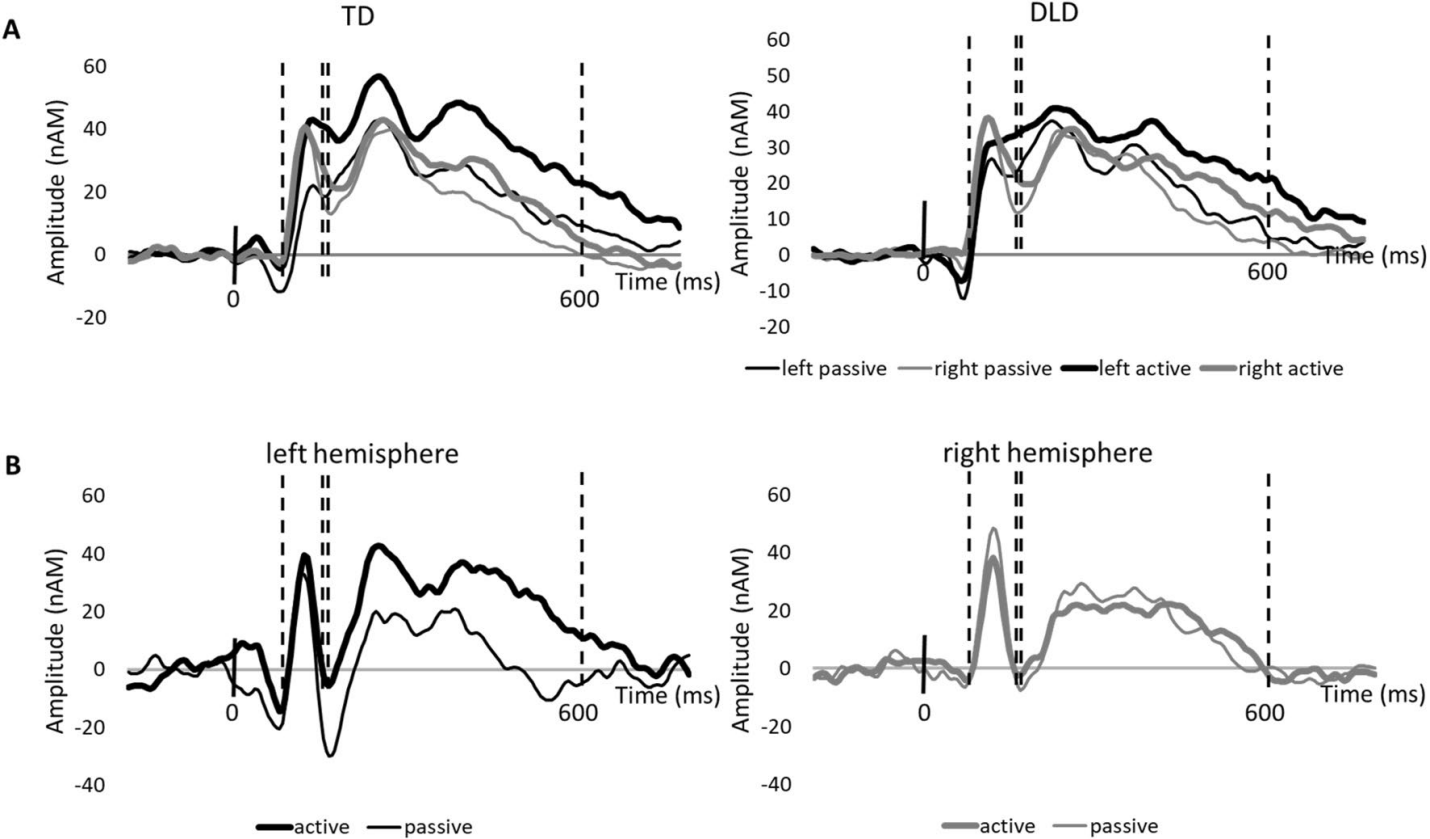
Time-course of activation of the dipolar sources. The early (90–180 ms) and late (185–600 ms) time windows are depicted with dashed vertical lines. (**A**) Grand-average waveforms of the ECDs located in the left (black lines) and right (gray lines) hemispheres separately for the TD and DLD groups. Responses to active and passive listening conditions averaged over the three sound types are plotted in thick and thin lines, respectively. (**B**) Average waveforms for active and passive listening in the left and right hemispheres of one TD participant.

Previous studies typically point to deficits in children with DLD either in the first^2,4,5,10^ or the second^6,8,27^ time window of auditory processing, as they were defined in the present study. Based on our findings, it seems relevant to acknowledge the dynamically varying nature of neuronal (auditory) processing for unbiased findings. For this, the temporal and spatial sensitivity of MEG offers an ideal means to approach even individual-level dynamics in the auditory cortex. However, the interindividual variance needs to be acknowledged when interpreting auditory-evoked responses. In our study, the large within-group variation in the amplitude values, especially in the first time window (cf. Figure 4A), aligns with earlier findings that allude to a strong impact of individual variability in the basic auditory response properties in children^22,42,43^ and adults^44^. This complicates the interpretation of group differences for this early time window. Indeed, particularly with small sample sizes, it is important to acknowledge the individual-level data, along with statistical testing.

It is however important to consider the limitations of the present study, mostly due to the small sample size. We believe this limitation to be mostly accounted for using linear mixed model approach, which allowed us to overcome the likely high differences in group variances. Although the use of this statistical test legitimates to spread our results to the similar population of children with DLD, our results should be interpreted with caution and suggest the need for replication with bigger sample sizes.

## Conclusion

This study showed an atypical interhemispheric balance during the auditory processing of speech and non-speech sounds in children with DLD, thus reinforcing the idea of approaching the deficit in DLD from the perspective of interhemispheric communication rather than as a hemisphere-specific dysfunction. Our findings further indicate the significance of the task requirements (active versus passive condition) for the appearance of auditory processing deficits in DLD. The abnormal interhemispheric asymmetry during passive auditory processing in children with DLD shifted toward the more typical left-lateralized response pattern in the active task condition. Further research is needed to elucidate the role of the task and the stimulation environment in determining hemisphere-specific engagement in different auditory conditions. Importantly, however, our findings evidence that the neural manifestation of top–down control seems to influence atypical brain reactivity to auditory input in DLD.

## Methods

### Participants

The participants of this study were selected from the cohort of a study aiming to investigate the etiology, linguistic development, and prognosis of DLD in the City of Vantaa, Finland^45,46^. As a result, all participants from the special education district of one municipality in the city of Vantaa were invited to participate in the study. Altogether, 18 participants volunteered. Nine of them were children with DLD (9–10 years old, six males). The other nine were children with typically developed (TD) language skills matched for gender and age. All participants were reported to be right-handed and native Finnish speakers. Individual hearing thresholds were tested to be within normal limits. The children with DLD had been diagnosed at Helsinki University Central Hospital prior to school entry.

Before starting the measurements, all the participants and/or their legal guardians signed an informed consent form in agreement with the prior approval of the Ethics Committee of the Hospital District of Helsinki and Uusimaa. The study was conducted according to good research practices, in line with legal requirements, and the guidelines of the Finnish National Board on Research Integrity.

### Stimuli and procedure

The stimuli were synthetic speech and non-speech sounds [cf.^47^]. Speech stimuli were utterances of the Finnish vowel /a/, created using a Klatt synthesizer^48^ for Macintosh (Sensimetrics, Cambridge, MA, USA). During the speech sound, the fundamental frequency (F0) decreased steadily from 118 to 90 Hz, resembling a normal male voice. The formant frequencies F1, F2, and F3 were 700, 1130, and 2500 Hz, respectively, and the formant bandwidths were 90 Hz for F1, 100 Hz for F2, and 60 Hz for F3. The vowel envelope had 15-ms fade-in and fade-out periods.

Non-speech stimuli were complex and simple sounds constructed based on speech sounds using Sound Edit (Macromedia, San Francisco, CA, USA). A complex sound was formed by a combination of three sine-wave tones, with the frequencies taken from the formants of a speech sound. A simple non-speech sound was a sine-wave tone composed of the same value of F2 frequency as in a speech sound. The envelope of a non-speech sound was similar to a speech sound. Each stimulus had a duration of 150 ms.

During the measurement, the participants were seated comfortably inside a sound treated magnetically shielded room and were instructed to avoid excessive movement of the head and eyes. Stimuli were controlled with the program Presentation (Neurobehavioral Systems Inc., San Francisco, CA) running on a PC and were delivered binaurally through plastic tubes and earpieces at 65 dB (SPL) above hearing level. The participants were instructed to listen to the stimuli in passive and active modes in separate sessions. During the passive condition (Fig. 2A), they watched silent cartoons and were instructed to ignore the sounds. During the active condition (Fig. 2B), the participants were asked to press a button, with their dominant hand, when they heard the same stimulus twice in a consecutive manner.

During the MEG recordings, stimuli were randomly delivered to the participants until a maximum of 100 stimuli per category was reached. The stimulation was stopped before when participants gave signs of being tired. In the active condition, a similar average number of stimuli were presented for DLD group (speech: 50,66; complex sound: 49; sine-wave tone: 47,11) and TD group (speech: 52,22; complex sound: 51,88; sine-wave tone: 52,22). In the passive condition, also a similar average of stimuli was delivered to the participants with DLD (speech: 63,88; complex sound: 58,12; sine-wave tone: 60,12) and controls (speech: 64,12; complex sound: 56,37; sine-wave tone: 58,5) in each category. Stimuli were separated by an interstimulus interval of two seconds. The total duration of the task was approximately 20 min per condition.

### MEG recordings and analysis

Magnetic brain responses were recorded using a 306-channel Elekta Neuromag™ Neuromagnetometer (Elekta Oy, Helsinki, Finland). Prior to the measurements, four head-position indicator coils were attached to the participants’ scalps, and the coil locations were determined with a 3-D digitizer in relation to three anatomical landmarks (nasion and pre-auricular points). At the beginning of each measurement, an electric current was applied to the coils to enable the measurement of their locations with respect to the MEG helmet. The head position within the helmet was tracked continuously throughout all measurements. Horizontal and vertical eye movements were also monitored using EEG electrodes (electro-oculogram [EOG] and electrocardiogram [ECG]).

The MEG signals were band-pass filtered at 0.1–200 Hz and sampled at 600 Hz. The raw data were pre-processed using the spatiotemporal signal space separation method (tSSS)^49^, included in Maxfilter software (Elekta, Neuromag), to compensate for possible head movements and to remove external interference emerging during the measurement. The initial head position was used as the destination head position for movement compensation. Epochs contaminated by eye movements (as measured with EOG) were rejected, and artifacts caused by cardiac signals (identified with the use of ECG) were suppressed by averaging the MEG signal with respect to heartbeat, using Principal Component Analysis (PCA) to identify the strongest components and removing the component(s) that captured the electromagnetic artifact produced by heartbeat^50^. Additionally, the data were visually inspected to exclude epochs contaminated by the remaining artifacts. The number of artifact-contaminated epochs eliminated were less than 2,5% of the total number of epochs. The artifact-free epochs were then averaged for intervals from 200 ms before stimulus onset (pre-stimulus baseline) until 800 ms after it.

The active neural populations were modeled using Equivalent Current Dipoles (ECD;^51^) with XFit software (Elekta Oy, Helsinki, Finland). Structural MR images were not available for these children. Therefore, an average sphere model for a group of children of the same age group who have been previously was used. For each participant, the center of the sphere was adjusted to the center of the head in the coordinates x =  − 2.0, y = 3.4, z = 44.4. The modeling was done by first selecting standard groups of 22 gradiometer sensors, one covering the left temporal area and the other covering the right temporal area (see Fig. 3A). The selected 22 sensors were the same across participants.

Separately in each hemisphere, an ECD was fitted within the time window of the robust evoked response at around 100 ms, based on the maximally dipolar topography and the maximum magnetic field strength. The well-known posterior-inferior current flow of the N100 response was confirmed in all the source models. Several candidate ECDs were fitted, and the one with the highest goodness-of-fit value was selected. The resulting ECDs were compared in terms of numeric goodness-of-fit value but also based on a visual match between measured and estimated signals. The goodness-of-fit value was the priority in choosing the final model, and in all cases, this also appeared to be the ECD with a good match between the measured and the estimated field pattern. Goodness-of-fit values were reasonable across all individuals (left hemisphere: on average 90.07, with minimum of 80 and maximum of 97.2; right hemisphere: on average 92.62, with minimum of 79.3 and maximum of 98).

Source modeling was performed separately for each participant. For every participant, ECDs were first determined separately for each stimulus. The locations and orientations of two ECDs, one for each hemisphere, were kept fixed, while their amplitudes were allowed to vary to best explain the signals recorded by all sensors over the entire averaging interval. The magnetic field topographies across sound types (speech, complex, and sine-wave tone) were comparable, and the same ECD successfully explained the activation for all sound types (see Fig. 4). Likewise, for most of the subjects, the active and passive conditions exhibited highly comparable spatial characteristics of activation. Therefore, to enable a reliable comparison of the strength of activation across experimental conditions, we used the same ECDs (identified in the active condition for speech, separately for left and right hemisphere) to extract the time course of activation for both active and passive conditions. As exemplified by Fig. 3B, the ECDs determined in the active condition accounted well the responses recorded in passive condition in both time-windows. For the early time-window the two-dipole model accounted for 73% of the measured data in the active condition, and 62% of the measured data in the passive condition. For the late time-window the two-dipole model accounted for 71% of the data in the active and 68% of the data in the passive condition.

Two-time windows of interest were defined for further analysis. These time windows were based on previous literature, where transient auditory activation was systematically observed within the initial 200 ms after the stimulus onset (N100m, [cf.^52,53^]), and a longer-lasting, developmentally specific response was reported after 200 ms (N250m, [cf.^22,29,47^]). The grand average time courses of the present data demonstrate robust and long-lasting activation throughout the first 600 ms (see Fig. 4A), and these activation components are clear, especially in the single-subject waveform (Fig. 4B). To quantify the strength of these activation components, the first time window was set to 90–180 ms (N100m) after the stimulus onset, and the maximum amplitude during this time window was collected separately for each stimulus type. The second time window comprised sustained brain activation at 185–600 ms after the stimulus onset. The mean amplitude value within this period was used in the analysis and collected separately for each stimulus type.

### Statistical analysis

When comparing a well-defined and homogeneous group (such as a clinical group) with a more randomly selected control group, as is the case here, the control group’s data may show more variability. Therefore, to consider both the correlations of repeated measurements within subjects and the possibly different variances between the DLD and TD groups, a linear mixed model (LMM) was used^54,55^. The LMM does not assume equal variances between groups; consequently, it is more suitable to handle the differences in variance between our groups.

Moreover, the 2 × 2 × 3 structure of the values for the brain responses in each individual (two attentional conditions per hemisphere for three types of sounds) leads to the non-independence of multiple responses from the same participant. In the LMM, this is considered using an individual-specific random intercept and slope (or slopes), leading to different baseline values and different regression coefficients at the individual level, respectively.

For the first time window, the test variable was the maximum amplitude within the time interval 90–180 ms post stimulation. For the second time window, the test variable was the mean amplitude from the interval of 185–600 ms after the stimulus onset. One model for each time window was computed using the fixed effects of hemisphere, sound type, group, attentional condition, and the random effects of a participant. The two variables (maximum and mean amplitude) were thus modeled separately by the candidate covariates: hemisphere (right/left, right as baseline), sound type (speech/sine/complex, speech as baseline), group (DLD/TD, DLD as baseline), and attention (passive/active, passive as baseline). The dependence between measurements can be modeled by adding a random intercept and slopes to a classical linear regression model at the individual level.

When selecting the model, a backward method was used, starting from the model with main effects, all pairwise interactions, a three-wise interaction, and a final random part. By testing the fixed effects, each model was estimated with a maximum likelihood (ML) method, and when testing random effects, a restricted maximum likelihood (REML) method was used. In the selection process, the nested models were compared using a likelihood ratio test and its *p*-value based on a chi^2^ distribution. Moreover, the difference in the variances of the group (DLD/TD) was tested using a likelihood ratio test. The final model was calculated using an REML to reduce the biases of estimated variances in the model. Besides the estimated fixed coefficients, a set of contrasts was formulated based on the final model and tested using t-tests or z-tests.

The validity of each mixed model was evaluated using graphical tools. The linearity was evaluated with a scatterplot of fitted values versus responses and a scatterplot of fitted values versus residuals separately for each group. The normality of the random effects and residuals was checked separately for each group using qq plots. The analysis was performed using R^56^ package nlme and its function lme.

## Data Availability

The dataset analyzed during the current study are not publicly available due to legal restrictions but are available from the corresponding author on reasonable request.
